# Journal of Comparative Medicine and Veterinary Archives for 1895

**Published:** 1894-12

**Authors:** 


### EDITORIAL.

### JOURNAL OF COMPARATIVE MEDICINE AND VETERINARY ARCHIVES FOR 1895.

The Journal of Comparative Medicine and Veterinary Archives for 1895 will be greatly strengthened in its various departments, and will, to the highest degree attainable, be replete with matters of interest to all veterinarians and students of comparative medicine. Records of all progressive movements in veterinary work at home and abroad will be carefully chronicled. Association news, National, State and local, will be regularly and promptly published, and all proposed legislation of a National State or local character will be kept before our readers. More original articles will be published than any year in our history, from the pens of the best writers at home and abroad. The veterinary and comparative medicine journals of the world will be drawn upon for choice selections and records of cases, treatment, etc., and thus give to our readers a rich summary of every advance in the domain of veterinary medicine and surgery. A complete record of college movements in North America will be a feature of the year, and every effort will be given by this journal to encourage and sustain an advanced movement in college work. A brief and fair review of all veterinary literature will be carefully given from time to time, and our readers will be kept thoroughly posted of all new books, etc. Veterinary sanitary work and the progress of animal industry will be kept carefully recorded.

News and items of interest will be published monthly, and, in fact, no feature of interest to the progressive veterinarian will be left untouched to make the Journal a welcome visitor to its every reader. Our columns will be always open for the records of cases, fair and gentlemanly criticism of all matters of public interest, and are freely extended to all our readers.

The price of the Journal will remain at $3.00 per year in advance, while special rates will be made to prospective and bona fide students at all veterinary or medical colleges.

Sample copies and all communications or inquiries will be cheerfully replied to. Address,

W. Horace Hoskins,
Business Manager,
3452 Ludlow Street, Philadelphia, Pa.

Rush Shippen Huidekoper, M.D., Veterinarian (Alfort),
155 West 56th St., N. Y. City.
W. A. Conklin, M.D., late Director Central Park Zoological Gardens, N. Y.,
51 Catharine St., N. Y. City.
A. W. Clement, V.S. (Montreal), late U. S. Inspector Bureau of Animal Industry,
916 Cathedral St., Baltimore, Md.
W. Horace Hoskins, D.V.S. (Am. Vet. College), President U.S. Vet. Med. Ass'n,
3452 Ludlow St., Philadelphia, Pa.
*Editors and Publishers.*

---

In this number of the Journal will be found a revised bill for the establishment of a Live Stock Sanitary Commission in Pennsylvania, together with an appeal for its favorable consideration, both timely and logical. Perhaps it may be said of this bill, without disparagement to any others, that while conservatively drawn, it is destined, if it becomes a law, to more satisfactorily solve the question of tuberculosis in our present light than any we have seen. It allows a wide latitude to the Commission, and does not surround its duties with doubtful specific instructions, and will thus not arouse in the minds of the laity rebellious feelings, and lead them to rear up obstacles in the way of its enforcement. We appeal to the veterinarians of Pennsylvania to work zealously for its enactment.

